# Binding of myeloperoxidase to the extracellular matrix of smooth muscle cells and subsequent matrix modification

**DOI:** 10.1038/s41598-019-57299-6

**Published:** 2020-01-20

**Authors:** Huan Cai, Christine Y. Chuang, Clare L. Hawkins, Michael J. Davies

**Affiliations:** Department of Biomedical Sciences, Panum Institute, University of Copenhagen, Copenhagen, Denmark

**Keywords:** Chemical modification, Oxidoreductases, Proteins, Post-translational modifications, Cardiovascular biology, Vascular diseases

## Abstract

The extracellular matrix (ECM) of tissues is susceptible to modification by inflammation-associated oxidants. Considerable data support a role for hypochlorous acid (HOCl), generated by the leukocyte-derived heme-protein myeloperoxidase (MPO) in these changes. HOCl can modify isolated ECM proteins and cell-derived matrix, with this resulting in decreased cell adhesion, modulated proliferation and gene expression, and phenotypic changes. Whether this arises from free HOCl, or via site-specific reactions is unresolved. Here we examine the mechanisms of MPO-mediated changes to human coronary smooth muscle cell ECM. MPO is shown to co-localize with matrix fibronectin as detected by confocal microscopy, and bound active MPO can initiate ECM modification, as detected by decreased antibody recognition of fibronectin, versican and type IV collagen, and formation of protein carbonyls and HOCl-mediated damage. These changes are recapitulated by a glucose/glucose oxidase/MPO system where low continuous fluxes of H_2_O_2_ are generated. HOCl-induced modifications enhance MPO binding to ECM proteins as detected by ELISA and MPO activity measurements. These data demonstrate that MPO-generated HOCl induces ECM modification by interacting with ECM proteins in a site-specific manner, and generates alterations that increase MPO adhesion. This is proposed to give rise to an increasing cycle of alterations that contribute to tissue damage.

## Introduction

The extracellular matrix (ECM) is essential for the homeostasis of tissues. In the case of the artery wall, the vascular basement membrane plays a key role in supporting and determining endothelial and smooth muscle cell survival and function^1^. This ECM forms a specialized and distinct microenvironment, with the biomacromolecules (e.g., collagens, laminins, fibronectin, perlecan, versican, elastin, nidogen, syndecans) that comprise this material, and a myriad of associated materials (e.g., cytokines, growth factors) dictating the shape and function of the artery wall, and the nature and behavior of the associated vascular cells^2^. Due to the lower turnover rate of many ECM materials (cf. estimated half-lives of 60–70 days and 74 years for arterial collagen and elastin, respectively^3,4^), and the low levels of antioxidant defense systems in extracellular environments^5^, ECM components and proteins in biological fluids appear to be particularly susceptible to oxidant damage. These factors result in a gradual accumulation of damage in the ECM with both aging and disease, including within the artery wall during the development of atherosclerosis (reviewed^2,6^). At least some of this damage appears to arise via the formation of oxidants generated by the leukocyte-derived heme enzyme myeloperoxidase (MPO)^7^.

Alterations to the structure and properties of ECM has been associated with the progression of atherosclerosis^8^. Previous studies have shown that approximately 70% of the oxidation products detected on proteins extracted from human atherosclerotic lesions are present on ECM proteins^7^, consistent with the high abundance of ECM in this tissue (estimated as 30–60%^9^). Multiple different oxidized protein side chains have been detected on lesion proteins at higher levels than in normal healthy tissue, with these including hydroxylated, cross-linked, nitrated and chlorinated species. Some of these products are associated with the presence and activity of particular oxidant generating systems, including redox-active metal ions (hydroxylated products)^10–12^, reactive nitrogen species (e.g. peroxynitrous acid, as evidenced by the presence of 3-nitrotyrosine and 6-nitrotryptophan)^13–16^, and reactive chlorine species such as hypochlorous acid (HOCl, generated by MPO) which generate 3-chlorotyrosine and other chlorinated products^17–22^. The relative roles of these oxidants, and the events that give rise to the observed damage remain to be fully established, though it is known that both reactive nitrogen species and HOCl can be generated by MPO (reviewed^23^).

Considerable evidence supports a role for MPO in the pathogenesis of atherosclerosis. MPO mRNA, protein and enzymatic activity are present in lesions^24^, elevated levels of HOCl-damaged proteins are present^18,25^, and increased concentrations of the HOCl-generated biomarkers, 3-chlorotyrosine and 5-chlorouracil have been reported^17,21^. There is a strong association between the levels of HOCl-damaged proteins and the severity of disease (as judged by intimal-medial thickness)^18,19^, and the plasma levels of MPO have been shown, in multiple epidemiological and clinical studies, to be both diagnostic and prognostic of poor cardiovascular (and other disease) outcomes^26–28^.

MPO is released from intracellular storage granules of activated neutrophils, monocytes and some tissue macrophages, and shows a high affinity for both ECM proteins and negatively-charged glycosaminoglycan (GAG) chains due to the high abundance of (positively-charged) Lys and Arg residues on the protein surface^29–31^. These observations suggest that the HOCl formation by MPO/H_2_O_2_/Cl^−^ may occur in a localized and site-specific manner. This is consistent with binding of (highly-cationic) MPO to ECM materials such as GAG chains^30,32^ including those of the glycocalyx^33^, proteoglycans such as perlecan^31^, and lipoproteins such as high- and low-density lipoproteins (HDL and LDL, respectively)^17,22,34^. MPO protein has been reported to be particularly localized to the shoulder regions of lesions, which are especially prone to rupture and thrombus formation^35,36^, and high numbers of MPO-expressing cells have been reported at such sites^37–39^.

In recent studies with isolated human plasma fibronectin exposed to HOCl or a MPO/H_2_O_2_/Cl^−^ system, differences were detected in both the extent and sites of modification between these two systems^40^. One potential explanation for these differences is site-specific formation of HOCl as a result of MPO binding to the protein^40^. As a consequence, we have examined the underlying mechanism(s) by which MPO-derived HOCl induces oxidation of human coronary artery smooth muscle cell (HCASMC) ECM, by examining the hypothesis that MPO binds to fibronectin and that this gives rise to site-specific oxidant formation and localized ECM damage.

## Results

### Decellularization of primary HCASMC cultures using sodium deoxycholate and immunolocalization of ECM components

Previous studies have commonly used NH_4_OH to remove cells from cultures to give isolated ECM preparations^41^. This method was used in initial experiments, but such treatment results in a loss of some ECM materials as judged by a decreased detection of fibronectin (determined by immunofluorescence using an anti-fibronectin pAb) in NH_4_OH-treated samples compared to controls (Supplementary Fig. 1).

**Figure 1:**
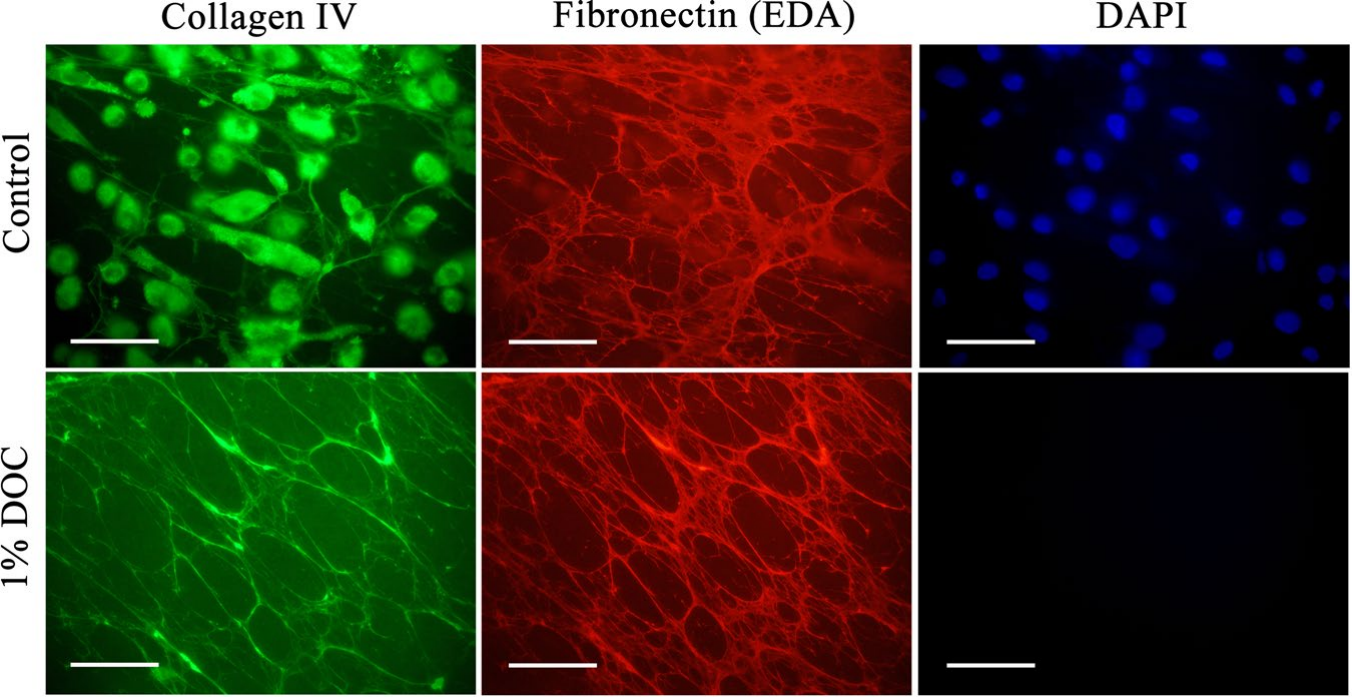
Immunolocalization of native and decellularized ECM components synthesized by primary HCASMCs. HCASMCs were cultured for 1 week to allow the synthesis and assembly of native ECM, then either left untreated (control) or treated with 1% sodium deoxycholate (DOC; bottom panel) to lyse and solubilize the attached cells. Matrix proteins were detected via double immunofluorescence staining using primary antibodies against type IV collagen (pAb) and the cell-derived fibronectin EDA region (FN-3E2). Alexa Fluor 488-conjugated anti-rabbit secondary antibody (green) and an Alexa Fluor 594-conjugated anti-mouse secondary antibody (red) were used to visualize matrix protein-primary antibody complexes, with nuclei counterstained using DAPI (blue). Representative images from n = 3 independent experiments are shown. Scale bars: 50 μm.

Recent studies have employed sodium deoxycholate (DOC, 1%) to decellularize murine tissues, with minimal reported damage to native ECM components, allowing high-resolution imaging and proteomic analyses^42^. Consequently, this treatment was examined as a potential method for obtaining native ECM from cell cultures. HCASMCs were cultured on chamber culture slides for 1 week to produce HCASMC-ECM. The slides were then examined either as intact structures, or incubated with 1% DOC to remove cells. Double immunofluorescence staining using primary antibodies against cell-derived fibronectin-extra domain A (EDA) (mAb 3E2) and type IV collagen (pAb) was then employed to examine both the presence and levels of ECM materials. The intact HCASMC-containing wells stained positive for type IV collagen both intracellularly and extracellularly (Fig. 1, top row; green fluorescence), cell-derived fibronectin localized extracellularly as fibre-like structures (Fig. 1, top row; red fluorescence), and cell nuclei (Fig. 1, top row; blue fluorescence from DAPI). Control samples showed no fluorescence indicating a high specificity of these antibodies (Supplementary Fig. 2). Examination of DOC-treated wells showed the presence of extracellular type IV collagen as fibre-like structures and cell-derived fibronectin but no blue fluorescence from DAPI (Fig. 1, bottom row). These data indicate that DOC treatment is an efficient and gentle decellularizing agent for monolayers of HCASMCs *in vitro*. Versican, another key matrix component, was also detected in the DOC treated cultures by double immunofluorescence staining using an anti-fibronectin pAb and anti-versican mAb (12C5, which recognizes the hyaluronate-binding G1 domain) (Supplementary Fig. 3).

### MPO binds to matrix proteins in HCASMC-ECM

**Figure 2:**
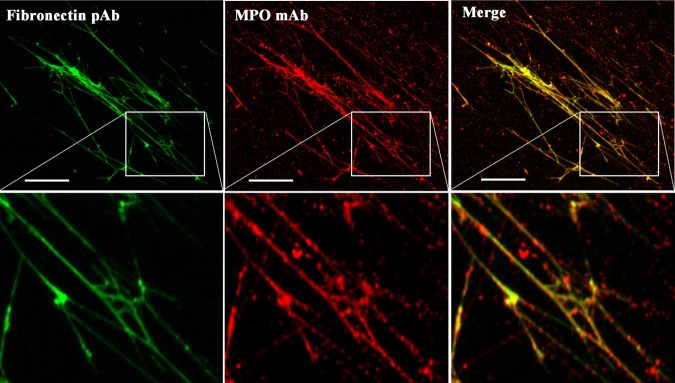
Colocalization of fibronectin and MPO in decellularized native HCASMC-ECM. Decellularized HCASMC-ECM was exposed to 20 nM of MPO at 37 °C for 30 min. Fibronectin and MPO were detected by primary antibodies: (left column) fibronectin pAb (A17), (middle column) anti-MPO 2C7 mAb. Merged images are shown in the right column. An Alexa Fluor 488-conjugated anti-rabbit secondary antibody (green) and an Alexa Fluor 594-conjugated anti-mouse secondary antibody (red) were used to visualize matrix protein-primary antibody complexes, by confocal microscopy. Representative images from n = 3 independent experiments are shown. Scale bars: 50 μm.

As MPO binds with high affinity to some biological materials (see Introduction) we hypothesized that MPO may bind to and colocalize with HCASMC-ECM materials and induce selective ECM damage. Consequently, we investigated the affinity of MPO for matrix proteins present in decellularized HCASMC-ECM. HCASMC-ECM samples were prepared using DOC, blocked with BSA, then incubated with MPO (20 nM) for 30 min at 37 °C. After washing to remove non-adherent materials, the presence of fibronectin and MPO was examined via double immunofluorescence using an anti-fibronectin pAb and an anti-MPO mAb (2C7). Fibronectin was detected within the decellularized HCASMC-ECM as fibre-like structures, with the fluorescence from MPO strongly co-localized with this material, as evidenced by the merged images (Fig. 2; see also Supplementary Fig. 2).

**Figure 3:**
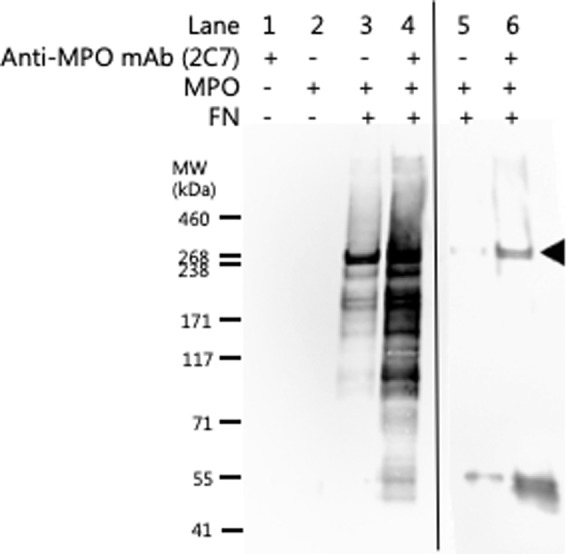
Immunoprecipitation of fibronectin using an anti-MPO mAb. Evidence for a direct MPO-FN interaction was investigated by immunoprecipitation using an anti-MPO mAb (2C7; 2 μg) bound to Protein A/G magnetic beads (Pierce, IL, USA). MPO (20 nM) was pre-incubated with FN (20 nM) to allow potential MPO-FN complex formation prior to incubation with anti-MPO mAb bound to magnetic beads. MPO-FN protein complexes immunoprecipitated with the anti-MPO mAb magnetic beads were separated on 3–8% tris-acetate SDS-PAGE gels and subsequently immunoblotted for the presence of FN monomers (filled arrow) using an anti-FN pAb and imaged by chemiluminescence. Lanes 1–4 were obtained from a blot with a long chemiluminescence accumulation time, carried out to confirm the absence of any recognized material in the controls represented by lanes 1 and 2. The detection of weak bands in lane 3 when compared to lane 4 indicates some non-specific binding of the FN-MPO complex to the beads alone. Lanes 5 and 6, are samples from another experiment, which used a shorter chemiluminescence detection time, to show the differences in staining intensity between the samples with and without mAb 2C7 against MPO. Representative images from n = 3 independent experiments are shown.

Further evidence for a direct MPO-FN interaction was obtained by immunoprecipitation of an MPO-FN complex using an anti-MPO mAb (2C7). The material pulled-down by mAb 2C7 showed positive staining at the expected mass of FN monomers, after separation of the proteins on SDS-PAGE and probing with an anti-FN pAb in immunoblotting experiments (Fig. 3).

### Modification of HCASMC-ECM proteins by MPO/H_2_O_2_/Cl^−^

**Figure 4:**
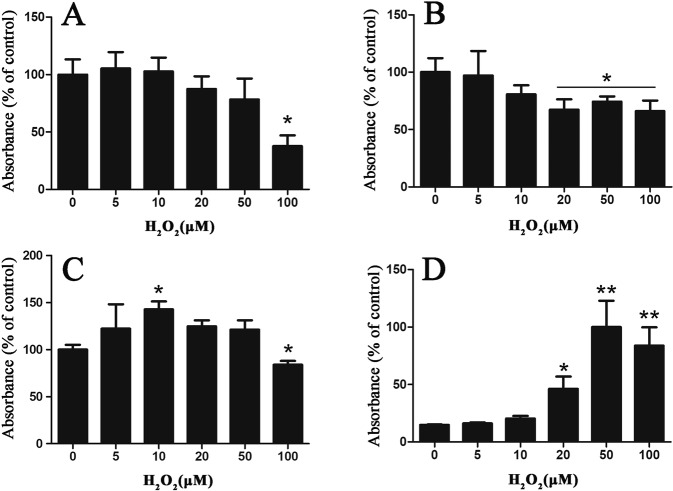
Effects of MPO-H_2_O_2_-Cl^−^ system on epitope recognition of primary antibodies against matrix proteins in native HCASMC-ECM. Decellularized HCASMC-ECM was left untreated (control; 0 μM) or treated with an MPO-H_2_O_2_-Cl^−^ system (20 nM MPO, 5–100 μM H_2_O_2,_ 200 mM Cl^−^). Loss of recognition of specific ECM components were detected by primary antibodies against: (**A**) fibronectin (pAb), (**B**) versican (12C5 mAb), (**C**) type IV collagen (pAb), and (**D**) the formation of HOCl-generated epitopes (mAb 2D10G9). Data are presented as means ± SEM from n = 3 independent experiments, and are expressed as a % of the control data (fibronectin, versican and type IV collagen), or the % of the maximal ELISA signal detected with mAb 2D10G9, with statistical analysis carried out using one-way ANOVA with Tukey’s post-hoc tests. * indicates statistical significance from the control (0 μM added H_2_O_2_) at the p < 0.05 level, and ** the p < 0.01 level.

We have shown previously that MPO-derived HOCl modifies HCASMC-ECM preparations decellularized using NH_4_OH^43^. Here, we investigated the extent of HOCl-induced modification generated on (more intact) DOC-decellularized ECM by a bound MPO/H_2_O_2_/Cl^−^ system, with alterations to the ECM detected by ELISA using primary antibodies that recognize HCASMC-ECM proteins. Native HCASMC-ECM was prepared using DOC (as above) and exposed to MPO (20 nM), Cl^−^ (200 mM) and varying concentrations of H_2_O_2_ (5–100 μM). This system gives near quantitative conversion of H_2_O_2_ to HOCl^40,43^. Exposure of the decellularized HCASMC-ECM to the MPO system with 100 μM H_2_O_2_ resulted in significant losses in epitope recognition by both the anti-fibronectin pAb (Fig. 4A) and anti-versican mAb (12C5), with ≥20 μM or higher H_2_O_2_ (Fig. 4B). With the type IV collagen pAb, a significant increase in epitope recognition was detected with 10 μM H_2_O_2_ (Fig. 4C), possibly as a result of increased exposure of cryptic epitopes as a result of modification to other ECM components. However, with 100 μM H_2_O_2_, a significant decrease in epitope recognition by this antibody was apparent. Concurrently, a significant increase in the presence of HOCl-generated epitopes was detected, as evidenced by recognition by mAb 2D10G9 (which recognises HOCl-modified proteins), with this being dose-dependent, and significant with ≥20 μM H_2_O_2_ (Fig. 4D).

**Figure 5:**
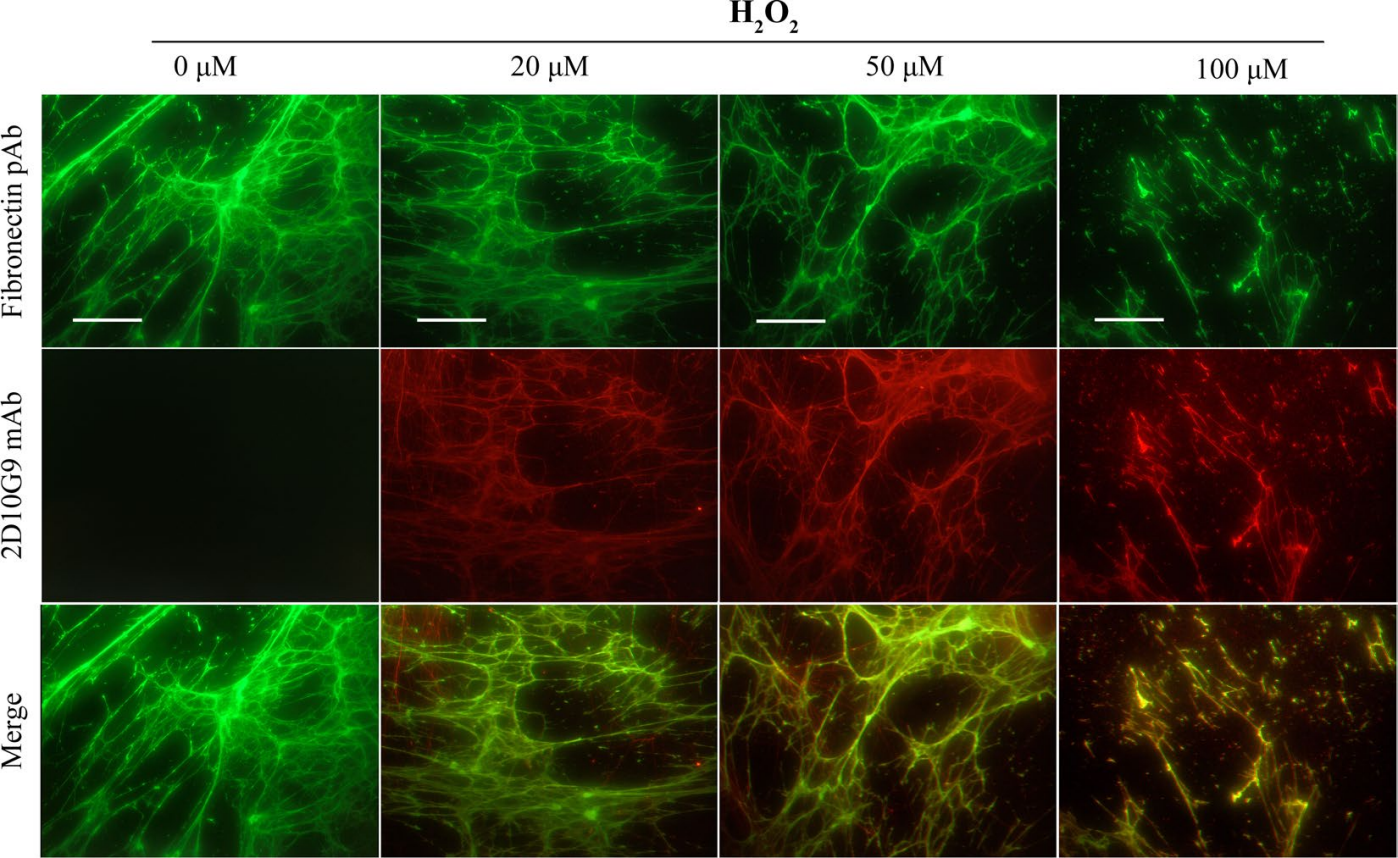
Effects of MPO-H_2_O_2_-Cl^−^ system on integrity of native HCASMC-ECM proteins. Decellularized native ECM was exposed to MPO-H_2_O_2_-Cl^−^ system (20 nM MPO, 0–100 μM H_2_O_2,_ 200 mM Cl^−^) at 37 °C for 2 h. The presence of HOCl-induced modifications to matrix proteins were detected via double immunofluorescence staining using fibronectin pAb (green) and mAb 2D10G9 (red), respectively. An Alexa Fluor 488-conjugated anti-rabbit secondary antibody (green, top row) and an Alexa Fluor 594-conjugated anti-mouse secondary antibody (red, middle row) were used to visualize matrix protein-primary antibody complexes. Merged images are shown in the bottom row. Representative images from n = 3 independent experiments are shown. Scale bars: 50 μm.

**Figure 6:**
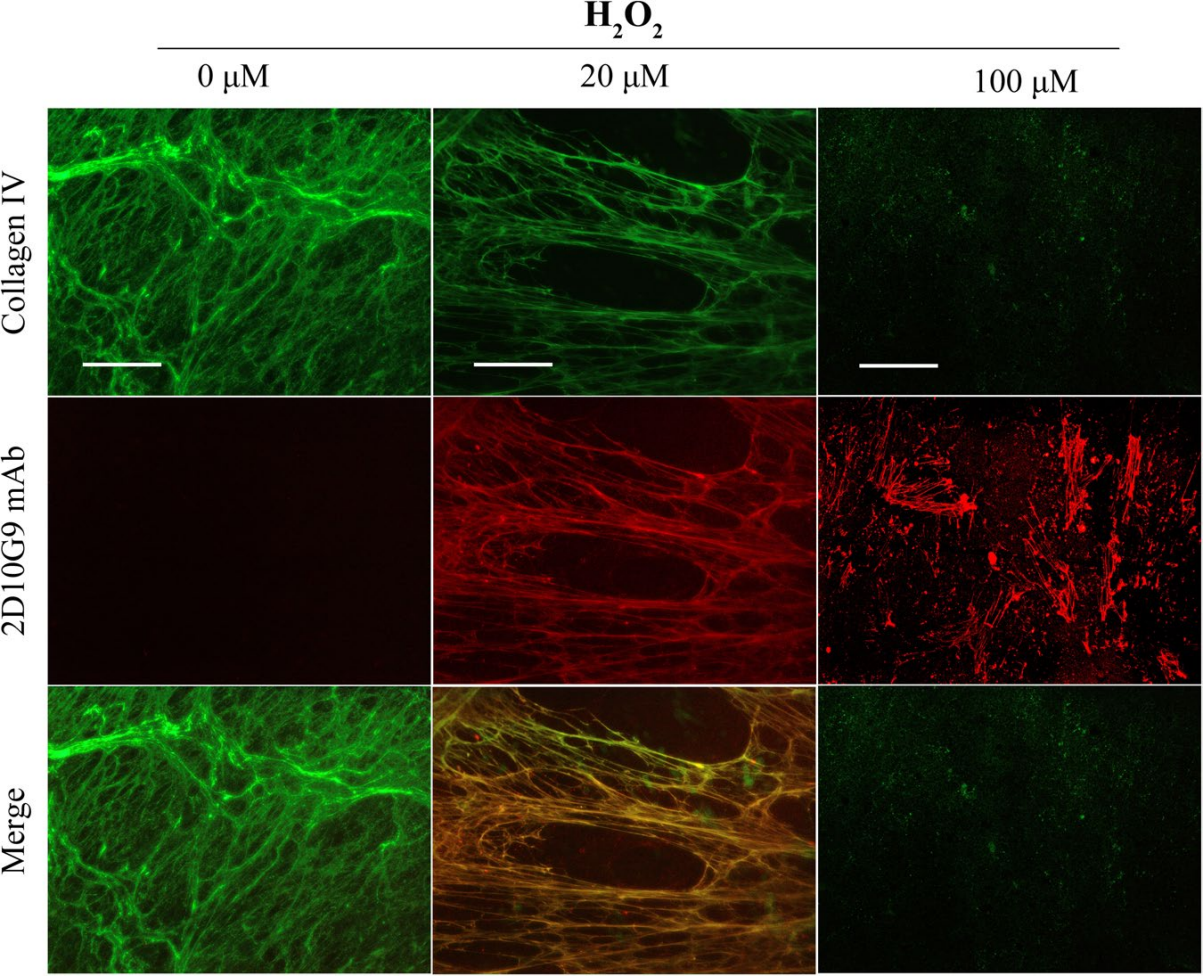
Effects of MPO-H_2_O_2_-Cl^−^ system on integrity of native Type IV collagen. Decellularized native ECM was exposed to MPO-H_2_O_2_-Cl^−^ system (20 nM MPO, 0–100 μM H_2_O_2,_ 200 mM Cl^−^) at 37 °C for 2 h. The presence of HOCl-induced modifications to matrix proteins were detected via double immunofluorescence staining using a collagen IV pAb and mAb 2D10G9, respectively. An Alexa Fluor 488-conjugated anti-rabbit secondary antibody (green, top row) and an Alexa Fluor 594-conjugated anti-mouse secondary antibody (red, middle row) were used to visualize matrix protein-primary antibody complexes. Merged images are shown in the bottom row. Representative images from n = 3 independent experiments are shown. Scale bars: 50 μm.

Increased recognition of ECM materials by the 2D10G9 mAb upon exposure to MPO/H_2_O_2_/Cl^−^ system was also examined using immunofluorescence staining. The fibronectin pAb, and mAb 2D10G9 were used to detect fibronectin and HOCl-generated epitopes in the decellularized HCASMC-ECM exposed to the MPO system, in experiments with 20–100 μM H_2_O_2_. These treatments resulted in significant damage to the matrix structure, as evidenced by fragmentation and loss of the fibronectin fibres with increasing H_2_O_2_ concentrations (Fig. 5, top row). Concurrent with these changes, increased recognition of matrix materials was detected with mAb 2D10G9 in a H_2_O_2_-dependent manner (Fig. 5, middle row). This staining colocalized with the fibronectin fibres (Fig. 5, bottom row). Similar data were obtained in immunofluorescence studies using a type IV collagen pAb and mAb 2D10G9, with a marked loss of recognition by the collagen antibody detected with both 20 and 100 μM H_2_O_2_ (Fig. 6, top row) and detection of material recognized by mAb 2D10G9 (Fig. 6, middle row), and colocalization of this fluorescence (Fig. 6, bottom row).

### Increased affinity of MPO for oxidized HCASMC-ECM

The above data are consistent with binding of MPO to HCASMC-ECM, with the MPO remaining catalytically-active and able to induce modifications to the ECM. However, it is unclear whether *oxidized* ECM also has this capacity. We therefore investigated the affinity of MPO for native, or HOCl-exposed HCASMC-ECM, via ELISA. Concurrently, the activity of matrix-bound MPO was examined in a quantitative manner using the TMB assay which relies on the conversion of taurine to taurine chloramine by HOCl, and subsequent assay of this chloramine^44^.

**Figure 7:**
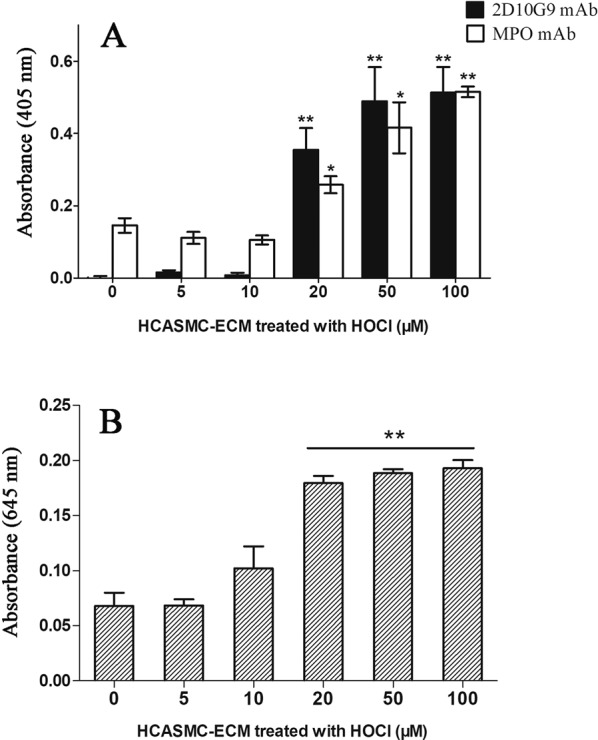
Increased affinity of MPO for oxidized HCASMC-ECM. (**A**) Decellularized HCASMC-ECM was prepared and exposed to buffer or 5–100 μM HOCl at 37 °C for 2 h, washed, and then incubated with 10 nM of MPO at 37 °C for 30 min. The samples were then washed to remove non-adherent material, before analysis of matrix-bound MPO and HOCl-mediated damage via ELISA using MPO mAb (2C7) and 2D10G9, respectively. In (**B**), matrix-bound MPO (prepared as described above) was incubated with 50 μM H_2_O_2_, 200 mM Cl^−^ and 10 mM taurine (to trap HOCl) at 37 °C for 2 h. The resulting taurine chloramines were assayed using TMB (see Materials and methods) with the absorbance recorded at 645 nm. Data from triplicate determinations (n = 3 independent experiments) were analyzed by one-way ANOVA with Tukey’s post-hoc test. * indicates statistical significance from the control (0 μM added HOCl) at the p < 0.05 level, and ** at the p < 0.01 level.

Reagent HOCl modified the decellularized HCASMC-ECM, with significantly increased mAb 2D10G9 recognition detected via ELISA, with this increasing in a dose-dependent manner; this increase was significant at ≥20 μM HOCl (Fig. 7A, black bars). Interestingly, an increased absorbance at 405 nm, arising from recognition by the MPO mAb, was also observed for the samples treated with ≥20 μM HOCl, and in a dose-dependent manner, consistent with MPO binding to the modified ECM, and having a higher affinity for the modified, compared to native, HCASMC-ECM (Fig. 7A, white bars). To confirm the increased binding of MPO to the oxidized ECM, the enzymatic activity of the matrix-bound MPO was quantified using the TMB assay, with a fixed concentration of H_2_O_2_ (50 μM) added to the MPO adherent on the ECM in the presence of taurine to trap the resulting HOCl. A significantly increased absorbance at 645 nm was observed for MPO bound to the ECM treated with 20 μM HOCl, when compared to the MPO bound to native ECM, consistent with an increased concentration of active MPO bound to the oxidized, compared to native, HCASMC-ECM (Fig. 7B). The yield of taurine chloramine quantified using TMB reached an apparent plateau when the initial ECM was treated with higher concentrations of HOCl, suggesting that the H_2_O_2_ added to the system can be depleted by the activity of the bound MPO.

**Figure 8:**
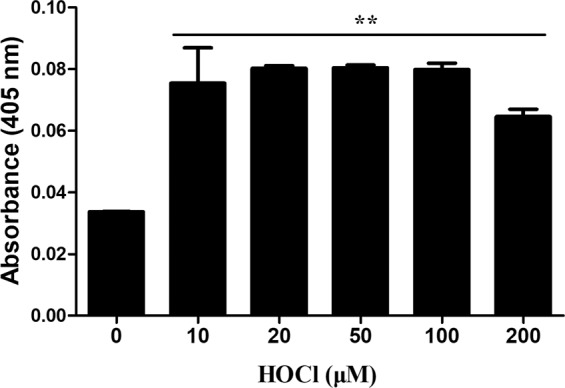
Formation of carbonyl groups on decellularized HCASMC-ECM by reagent HOCl. Decellularized HCASMC-ECM was prepared then either left untreated (control; 0 μM) or treated with reagent HOCl (0–200 μM) for 2 h at 37 °C before determination of the protein carbonyl content after derivatization with 2,4-dinitrophenylhydrazine, and subsequent recognition of the corresponding hydrazone by a commercial antibody with ABTS as the chromophore. Data from n = 3 independent experiments are expressed as mean ± SEM of the absorbance values at 405 nm. Statistical analysis was carried out using one-way ANOVA with Tukey’s post-hoc tests. ** indicates significance at the p < 0.01 level.

The potential role of ECM oxidation products in the increased affinity of MPO for the oxidized matrix, was examined by quantifying the level of carbonyls formed on the HCASMC-ECM treated with different concentrations of reagent HOCl (10–200 μM). The carbonyl yields were measured by derivatization with 2,4-dinitrophenylhydrazine (DNPH) and detection of the resulting hydrazone products using an anti-DNPH antibody (Fig. 8). Treatment with HOCl resulted in a marked increase in HCASMC-ECM-derived carbonyl levels even at the lowest oxidant levels tested (10 μM), though the levels of these products did not increase in a linear manner with increasing HOCl concentrations, consistent with further reaction of these (reactive) products. Such behavior is consistent with previous reports^45,46^.

### MPO-induced HCASMC-ECM oxidation is dependent on the flux of H_2_O_2_

The data presented above indicate that a MPO-mediated oxidation system can modify decellularized HCASMC-ECM, as detected by ELISA or immunofluorescence staining with mAb 2D10G9. This occurs in a concentration-dependent manner with increasing concentrations of H_2_O_2_ ≥ 20 μM. Decreased ECM epitope recognition was also detected by antibodies raised against fibronectin, type IV collagen and versican mAb (12C5). However, the H_2_O_2_ used in these experiments was added as a bolus dose rather than continuously, with the latter more likely to reflect the biological situation. The overall concentrations are however close to those reported as a steady-state level (cf. reports of H_2_O_2_ concentrations in plasma of 1–10 μM^47^). As a consequence, we examined modification of the HCASMC-ECM by associated MPO where the H_2_O_2_ was generated *in situ* using a glucose/glucose oxidase (GO) system, that generates H_2_O_2_ over a long time period, with potential damage to the HCASMC-ECM detected using immunofluorescence staining and ELISA, as described above.

**Figure 9:**
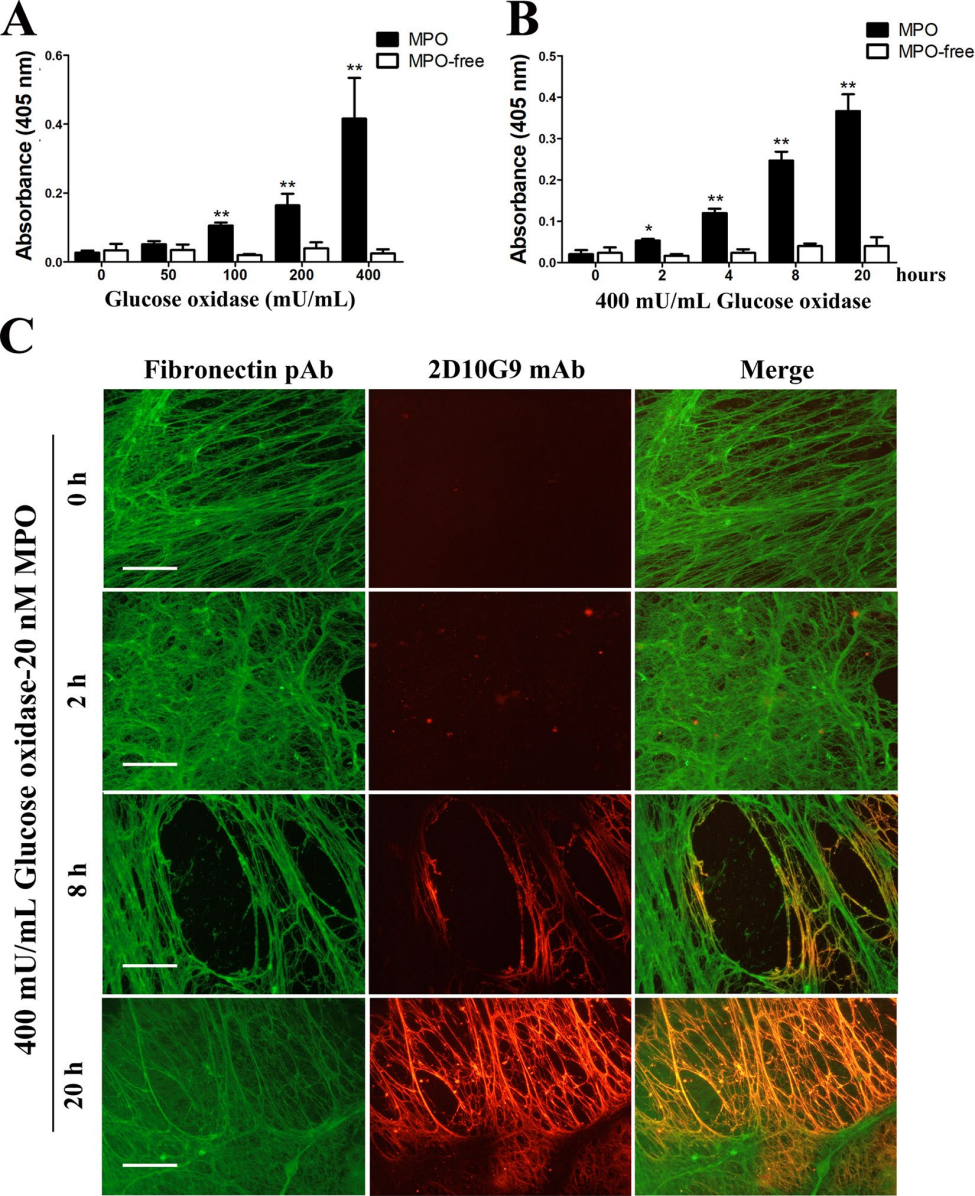
MPO-induced HCASMC-ECM oxidation is dependent on the flux of H_2_O_2_. HCASMC-ECM was incubated with (**A**) GO-MPO system (0–400 mU mL^−1^ glucose oxidase, 5 mM glucose, 20 nM MPO) for 20 h, or (**B**) GO-MPO system (400 mU mL^−1^ glucose oxidase, 5 mM glucose, 20 nM MPO) for 0–20 h. The extent of ECM damage was then quantified using the mAb 2D10G9 in an ELISA assay. (**C**) HCASMC-ECM was exposed to a GO-MPO system (400 mU mL^−1^ glucose oxidase, 5 mM glucose, 20 nM MPO) for 0–20 h, with parent ECM fibronectin and oxidative modifications to the HCASMC-ECM detected using immunofluorescence. Fibronectin and HOCl-induced modifications were detected using fibronectin pAb (left column) and mAb 2D10G9 (middle column), the corresponding images are merged in the right column. An Alexa Fluor 488-conjugated anti-rabbit secondary antibody (green) and an Alexa Fluor 594-conjugated anti-mouse secondary antibody (red) were used to visualize matrix protein-primary antibody complexes. In (**A** and **B**), absorbance data at 405 nm are presented as means ± SEM from n = 3 independent experiments. * Indicates statistical significance from the control (0 μM added GO or time zero data) at the p < 0.05 level, and ** at the p < 0.01 level by ANOVA with Tukey’s post-hoc tests. In (**C**), representative images from n = 3 independent experiments are shown. Scale bars: 30 μm.

The GO/glucose/MPO/Cl^−^ system generated oxidative damage to HCASMC-ECM in a GO concentration-dependent manner as determined by ELISA, with GO levels ≥100 mU mL^−1^ giving a statistically-significant increase in damage (Fig. 9A, black bars) relative to the corresponding controls that did not contain MPO (Fig. 9A, white bars). Exposure of HCASMC-ECM to a fixed concentration of the oxidant system (400 mU mL^−1^ GO, 5 mM glucose, 20 nM MPO, 200 mM Cl^−^) for increasing incubation times gave a time-dependent increase in HCASMC-ECM damage, as evidenced by increasing ELISA signals arising from mAb 2D10G9, for exposure times ≥2 h (Fig. 9B, black bars). In the absence of MPO this increase in absorbance was not detected (Fig. 9B, white bars). Immunofluorescence staining using the pAb against fibronectin, and mAb 2D10G9 against HOCl-mediated damage, supported this conclusion. Thus, significant green fluorescence arising from the presence of fibronectin was observed in each group (Fig. 9C, left column), whereas red fluorescence arising from the presence of HOCl-modified epitopes was absent in the controls, but increased in extent with longer incubation times (Fig. 9C, middle column), such that a marked increase in fluorescence was detected after 20 h. The fluorescence from these two antibodies was strongly colocalized (Fig. 9C, right column).

## Discussion

It is well established that elevated levels of oxidized DNA, RNA, proteins and lipids are present in atherosclerotic lesions, but the nature of the oxidants that generate these changes and the specific pathways involved remain to be fully elucidated^48^. We have reported that exposure of HCASMC-ECM to either reagent or MPO-derived HOCl can give rise to oxidative modifications of the HCASMC-ECM and its components, and that these modified materials affect the behavior of HCASMCs, including loss of adhesion, accelerated proliferation and modulated gene expression^43^. While MPO is vital for innate immunity^49^, aberrant expression of MPO and subsequent oxidant production have been implicated in many inflammatory diseases, including atherosclerosis (ref. ^36^, see also Introduction).

Here we have examined, at an *in vitro* level, how MPO modifies matrix proteins. MPO is shown to bind to ECM proteins, and to retain its catalytic activity, resulting in the generation of HOCl in the presence of Cl^−^ and H_2_O_2_, resulting in modifications to HCASMC-ECM materials. These modifications are localized to the vicinity of MPO, though the exact targets and specific binding sites require confirmation. These changes were recapitulated by exposure of HCASMC-ECM to a GO/glucose/MPO/Cl^−^ system, as evidenced by increased antibody recognition of HOCl-mediated damage (via ELISA and immunofluorescence assay) by 2D10G9 mAb. This occurred in a time-dependent manner, with increasing levels detected with higher fluxes of H_2_O_2_ as judged by a dependence on the GO concentration. A previous study has also reported direct modification of isolated fibronectin by H_2_O_2_, but this was dependent on trace metal ions which generate hydroxyl radicals (HO^.^) and required high levels of H_2_O_2_^50^. Whether such HO^.^ mediated damage is of importance *in vivo* remains to be established, as redox active metal ions would be expected to be tightly-bound to endogenous metal-ion binding proteins.

Interestingly, the extent of MPO-binding appears to be dependent on the level of HOCl-induced modifications, with greater binding apparent (when compared to the non-treated HCASMC-ECM) after pre-treatment of the ECM with HOCl, as indicated by the ELISA data where increased epitope recognition of MPO by anti-MPO mAb (2C7) was observed with HOCl-pre-treated HCASMC-ECM. Our data are therefore consistent with a new paradigm in which MPO participates in the development and progression of atherosclerosis through the generation of damage to matrix proteins, which then bind increasing concentrations of MPO as a result of an enhanced affinity of the enzyme for modified ECM. This bound MPO has been shown to retain a high level of enzymatic activity. This would result in a vicious cycle of increasing ECM alteration by MPO-derived oxidants, greater MPO binding to the damaged materials, and increased site-dependent oxidant formation. This has been associated with aberrant cell behavior including decreased cell adhesion, modulated proliferation, phenotypic switching of smooth muscle cells, altered gene expression, release of pro-inflammatory species, and matrix remodeling^43^.

The cultured primary HCASMC employed in this study produce abundant ECM materials *in vitro*, which in turn support the adhesion and survival of adherent cells. Immunofluorescence staining shows that different matrix proteins, including fibronectin, type IV collagen and versican, appear as fibre-like structures, indicating that these ECM proteins interact with each other to form a 3-dimensional scaffold to which the HCASMCs adhere and proliferate. We have previously prepared decellularized HCASMC-ECM using NH_4_OH^43^, which allows efficient removal of adherent cells, but this protocol has been shown here to result in loss and disruption of the ECM materials and structure. However, DOC, which has been successfully used to decellularize other tissues^42^, allows the harvesting of matrix components laid down by HCASMC, which appear to be structurally-intact. This intact decellularized HCASMC-ECM provides a better model to investigate matrix-cell and matrix-MPO interactions *in vitro*.

The current data indicate that MPO binds to matrix proteins, as evidenced by the colocalization of fibronectin and MPO detected via double immunofluorescence staining, confirming the data of Kubala *et al*.^30^. This suggests that MPO-induced damage to matrix proteins may be dependent on the specificity of binding to matrix proteins or sites. This is supported by the immunofluorescence staining, which shows that the HOCl-oxidized epitopes were primarily detected in the same regions as MPO binding to the ECM materials.

The mechanism(s) that underlie the increased MPO binding to HOCl-modified ECM have not been determined in detail, but the results obtained, and literature data provide a potential explanation. It is known that HOCl preferentially targets Cys, Met, cystine, His, α-amino, Trp, Lys and Tyr residues, in order of decreasing reactivity^51,52^. The resulting products have been elucidated^53^. For some residues, reaction results in conversion of a neutral species to a negatively-charged product (e.g. conversion of Cys and cystine to oxy acids, oxidation of His to give the carboxylic-acid containing residue Asp)^53^. Reaction at other residues (e.g. at His, α-amino and Lys) converts positively-charged (protonated nitrogen) groups to neutral species (e.g. Asn from His, carbonyls and nitriles from α-amino and Lys species)^53^. This increased negative charge, and loss of positive charge, may result in increased binding of (highly-positively charged) MPO via electrostatic interactions. Covalent bonding may also contribute, as elevated levels of carbonyls (a known product of amine (Lys) oxidation by HOCl^45^), were detected on HOCl-treated HCASMC-ECM. Carbonyls can undergo Schiff base reactions with Lys and Arg residues, that are present in abundance in the MPO structure (55 Arg and 14 Lys in the mature protein, ~12% abundance: UniProt entry: P05164). Such reactions have been reported previously to contribute to inter-molecular protein cross-linking (e.g.^54^).

The MPO bound to oxidized HCASMC-ECM is enzymatically active, as shown by the detection and quantification of taurine-derived chloramines. Whether this bound material is more or less active than free MPO, is unclear and difficult to assess due to difficulties in quantifying the amount of MPO bound to the ECM. Recent mass spectrometry data suggests that some ECM proteins are less extensively modified by the MPO system relative to the same concentration of reagent HOCl (e.g. fibronectin^40^), and others such as laminins more modified^55^, as judged by both the number, and level of modifications. These differences have been rationalized in terms of MPO binding to these species and possible alterations in activity. In HCASMC-EMC both fibronectin and laminins are present, therefore it is not possible to decipher the which effect may be more important. Although the nature of the specific MPO binding sites remain to be elucidated, it is nonetheless clear that higher levels of MPO are bound to the ECM after prior matrix oxidation.

These data imply that *in vivo*, increased inflammation, enhanced neutrophil/monocyte/macrophage activation and release of MPO, may result in increasing levels of MPO being associated with the ECM, which could promote further matrix modification. This positive feedback loop could potentially increase the rate of disease development and result in the accumulation of oxidized ECM proteins. This is consistent with the detection of elevated levels of oxidized materials in advanced human atherosclerotic lesions compared to healthy tissues, and an accumulation with age and disease^7^. The low turnover rate and poor antioxidant activity of the ECM^5^ would also be expected to favor such accumulation.

**Figure 10:**
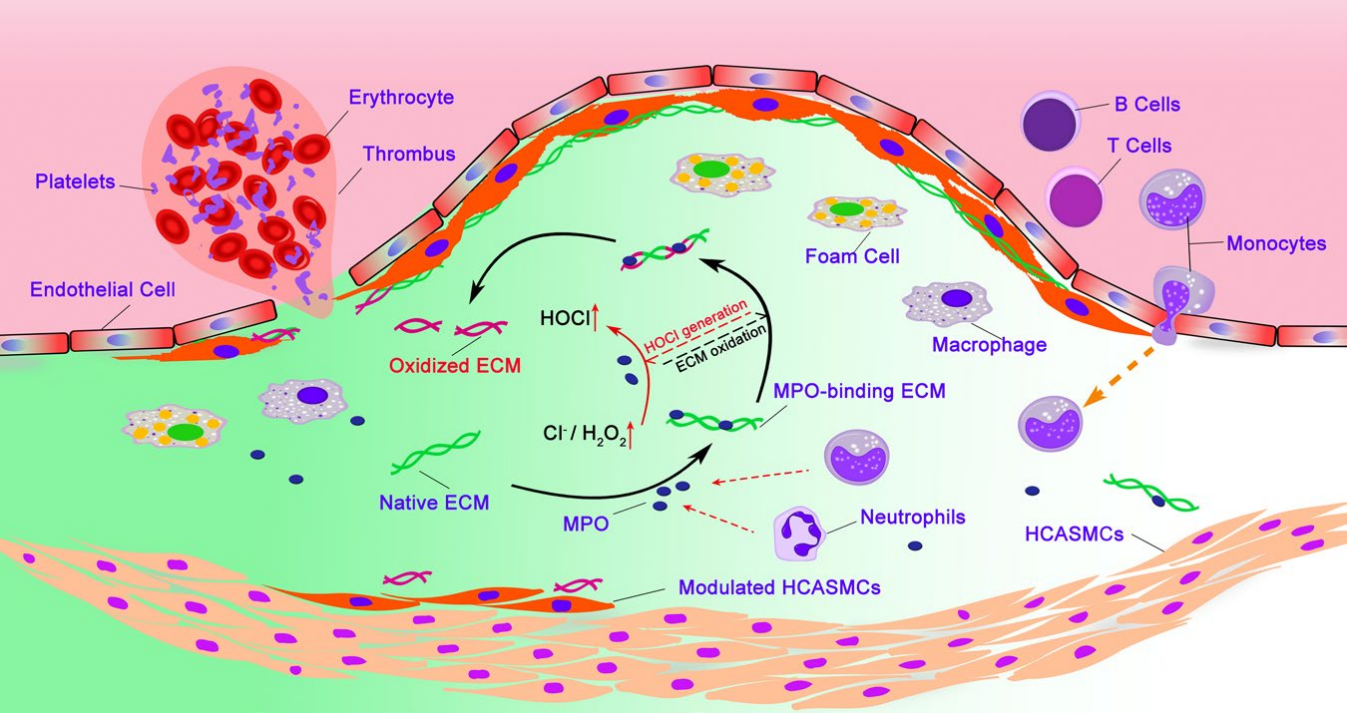
Diagram summarizing proposed mechanism by which MPO induces matrix oxidation and its contributions to the generation of vulnerable plaques. Myeloperoxidase (MPO) released by activated leukocytes shows a high affinity for matrix proteins, with its catalytic activity remaining unchanged. Matrix-bound MPO catalyzes the reaction of H_2_O_2_ with Cl^−^ to give HOCl, which subsequently induces oxidation of matrix proteins to which MPO is bound or closely associated. The progression of atherosclerosis enhances the concentration of H_2_O_2_ within the lesion, which facilitates the accumulation of oxidative modifications to the ECM of the lesion, increasing the susceptibility of the lesion to further damage and rupture.

Overall our data demonstrates, at an *in vitro* level, a mechanism by which MPO induces and drives increasing modification of the ECM (Fig. 10). MPO appears to have a high affinity for at least some matrix proteins, with the matrix-bound MPO able to catalyse HOCl generation, and oxidative modifications to the ECM; this appears to be localized at, or near, sites of MPO-binding. These modifications then enhance further binding of MPO and damage, with this accumulating in a time- and HOCl flux-dependent manner. These data increase our understanding of the mechanisms that underlie MPO-matrix interactions and MPO-induced matrix oxidation, and provide potential insights into the prevention or amelioration of inflammation-associated diseases, including atherosclerosis.

## Materials and Methods

### Reagents

All solutions were prepared using nanopure Milli-Q H_2_O (Millipore). H_2_O_2_ and HOCl concentrations were quantified spectrophotometrically (H_2_O_2_, ε_240 nm_ 43.6 M^−1^ cm^−1^ ^56^; HOCl (at pH 12), ε_292 nm_ 350 M^−1^ cm^−1^ ^57^). pH control of reaction mixtures was achieved by using 0.1 M sodium phosphate buffer, pH 7.4. All chemicals were obtained from Sigma (Sigma-Aldrich) unless otherwise stated. Human polymorphonuclear leukocyte-derived MPO was purchased from Planta Natural Products (Vienna, Austria).

### Cell culture of HCASMC

Commercially-sourced primary HCASMCs (Cell Applications, San Diego, USA; donor number #1596, Caucasian, male, age 53) were cultured (up to passage 5) in T-75 tissue culture flasks under 20% O_2_ and 5% CO_2_, at 37 °C in a humidified incubator. Cells were seeded at an initial density of 5 × 10^5^ cells in 15 mL growth medium (Cell Applications, USA; Smooth Muscle Cell Growth Medium, product number #311-500), with the media replaced three times per week.

### Immunofluorescence staining of ECM materials and modification products

HCASMCs (passage 4) were seeded onto eight-well chamber glass culture slides (Becton Dickinson) at an initial density of 2 × 10^4^ cells per well and cultured for 1 week to allow ECM synthesis. HCASMCs on the slides were treated twice with or without 1% (w/v) sodium deoxycholate (DOC) for 10 min at 21 °C with gentle rocking, followed by 5 washes with PBS. The slides were then fixed with 4% (v/v) formaldehyde at 37 °C for 15 min, then permeabilized using 0.5% (v/v) Triton X-100 in PBS on ice for 5 min. The slides were blocked with 1% (v/v) BSA in PBS at 21 °C for 1 h and then incubated with primary antibodies at 4 °C overnight. The primary antibodies were used at the following dilutions: anti-cell derived fibronectin EDA epitope (Sigma, 3E2 mAb; 1:1000), anti-fibronectin pAb (Abcam, ab2413; 1:1000), anti-type IV collagen (Abcam, ab6586; 1:1000), anti-MPO (Abcam, 2C7 mAb; 1:1000), anti-versican G1 domain (Developmental Studies Hybridoma Bank, Univ. of Iowa, USA, 12C5 mAb; 1:1000) and anti-HOCl generated epitopes (kindly provided by Prof. Ernst Malle, Medical University of Graz, Austria; 2D10G9 mAb, 1:200). The slides were rinsed twice with PBS then incubated with anti-mouse IgG conjugated with Alexa Fluor 488 antibody and anti-rabbit IgG conjugated with Alexa Fluor 594 antibody (both Molecular Probes; 1:1000 in 1% (v/v) BSA in PBS) at 21 °C for 1 h. The slides were rinsed again with PBS and then counterstained with 1 μg mL^−1^ DAPI (4′,6-diamidino-2-phenylindole, Molecular Probes) in PBS in the dark at 4 °C for 10 min, followed by three rinses with PBS, before being air dried and the cover slips added, with imaging using a fluorescence microscopy (Olympus, Japan) equipped with cellSense Entry v1.5 software.

### ELISA of ECM materials and modification products

HCASMCs were cultured in 96-well plates at an initial density of 1 × 10^4^ cells per well in 200 μL of growth medium (as above) for 1 week to allow synthesis of native HCASMC-ECM. The plates were rinsed twice with PBS followed by the incubation with 1% sodium deoxycholate (DOC) twice for 10 min at 21 °C. The plates were rinsed 5 times with PBS and the prepared native HCASMC-ECM was then treated with 50 μL of HOCl (0–100 μM), or an MPO-H_2_O_2_-Cl^−^ system (20 nM MPO, 0–100 μM H_2_O_2_, 200 mM Cl^−^) at 37 °C for 2 h. Alternatively, a glucose oxidase (GO)-MPO system was used to treat HCASMC-ECM. Briefly, the HCASMC-ECM in 96-well plates were prepared as described above and then incubated with either 50 μL of GO-MPO system (0–400 mU mL^−1^ GO and 20 nM MPO) for 20 h at 37 °C, or with 50 μL of GO-MPO system (400 mU mL^−1^ GO, 20 nM MPO) for 0–20 h at 37 °C. The plates were rinsed twice with PBS followed by blocking with 0.1% (w/v) casein in PBS. Plates were then incubated with the following primary antibodies, at the indicated dilutions, at 4 °C overnight: anti-fibronectin pAb (1:1000), anti-versican G1 domain mAb (12C5; 1:1000), mouse anti-MPO mAb (2C7, 1:1000) and anti-HOCl generated epitope mAb (2D10G9; 1:200). For the experiments using HOCl, the plates were rinsed twice with PBST (0.1% Tween-20 in PBS) and incubated with either horseradish peroxidase (HRP)-conjugated sheep anti-mouse or anti-rabbit IgG secondary antibodies (1:1000) followed by two more washes with PBST. The plates were subsequently developed with 50 mM sodium citrate (pH 4.6) containing 2 mM ABTS [2,2′-azinobis-(3-ethylbenzothiazoline-6-sulfonic acid] and 3 mM H_2_O_2_ for 30 min. For the experiments using MPO, the plates were incubated with alkaline phosphatase-conjugated rabbit anti-mouse (Abcam) or goat anti-rabbit IgG (Abcam) secondary antibodies at 21 °C for 1 h, washed twice with PBST then developed with *p*-nitrophenylphosphate solution (Merck) at 21 °C for 20 min. The optical absorbance of the resulting chromophores was determined using a Spectra Max i3x microplate reader (Molecular Devices). The alkaline phosphatase system was employed for MPO experiments, rather than the HRP/ABTS/H_2_O_2_ system, as both MPO and HRP react with H_2_O_2_, which causes erroneous absorbance data. Values for 1% BSA/PBS only wells were used as background to normalize the ELISA readings.

### Immunoprecipitation (IP) of MPO-FN protein complexes

MPO (20 nM) was incubated with human plasma-derived FN (20 nM) in PBS for 30 min at 37 °C to generate possible MPO-FN complexes. Protein A/G magnetic beads (25 μL per IP sample; Pierce, IL, USA) were prepared as per the manufacturer’s instructions, and incubated with 2 μg of anti-MPO mAb (2C7) for 1 h at 21 °C on a rotating mixer. The beads were then washed twice with the wash buffer provided by manufacturer, and in accordance with the provided instructions, prior to incubating with the MPO-FN mixture overnight at 4 °C on the rotating mixer. Negative controls using beads only with MPO/anti-MPO mAb (2C7) or MPO-FN complexes alone were also included. Next day, the beads were washed twice with wash buffer and once with MilliQ water prior to the addition of 20 μL elution buffer and 2 μL of neutralization buffer for 10 min at 21 °C. The eluted samples were transferred to clean tubes and 8.5 μL of 4x NuPAGE sample buffer, and 3.4 μL of 10x NuPAGE reducing agent, were added then heat denatured for 5 min at 90 °C as per the manufacturer’s protocol (Invitrogen, CA, USA). Seventeen μL of sample were loaded per lane, on to 3–8% Tris-acetate gels and electrophoresed for 65 min at 160 V. The separated proteins were then transferred onto a PVDF membrane using an iBlot 2 system (Invitrogen, CA, USA) for 7 min at 25 V. The membrane was then blocked using 1% (w/v) BSA/TBST (0.1% Tween-20) blocking buffer for 1 h, at 21 °C, then incubated for 1 h with anti-FN pAb (1:2000 dilution; ab2413; Abcam) on a shaker at 21 °C. The membrane was washed twice with TBST prior to incubating with donkey anti-rabbit IgG HRP conjugated (1:5000 dilution) secondary antibody for 1 h at 21 °C followed by 4 × 15 min TBST washes. The membrane was rinsed twice with TBS and subsequently imaged using a Sapphire Azure biomolecular imager (Azure Biosystems, CA, USA) to examine the presence of MPO-FN protein complexes immunoprecipitated using anti-MPO mAb (2C7).

### Quantification of HOCl production by reaction with taurine and assay with 3,3′,5,5′-tetramethylbenzidine (TMB)

Decellularized HCASMC-ECM in 96-well plates was prepared as described above. The plates were incubated with 50 μL of HOCl (0–100 μM) at 37 °C for 2 h followed by two rinses with PBS. Plates were then blocked with 1% BSA/PBS at 21 °C for 1 h and then incubated with 20 nM of MPO at 37 °C for 30 min. Residual materials were then removed by washing twice with PBS, followed by incubation with 50 μM H_2_O_2_, 200 mM Cl^−^ and 10 mM taurine at 37 °C for 2 h. The HOCl generated by matrix-bound MPO, and trapped with taurine as the corresponding chloramine, was subsequently quantified^44^ using a developing reagent consisting of 20 mM TMB in dimethylformamide and 2 mM sodium iodide in sodium acetate buffer (0.44 M, pH 5.4). This was prepared immediately prior to addition to all samples, with 25 μL added to each well and incubated at 21 °C for 5 min. The absorbance at 645 nm was subsequently determined using a Spectra Max i3x microplate reader (Molecular Devices).

### Carbonyl assay

The plates for carbonyl detection were prepared as described above for the ELISA experiments. The resulting native HCASMC-ECM was exposed to 50 μL of HOCl (0–200 μM) for 2 h at 37 °C and the plates were then rinsed twice with PBS. The carbonyl levels were quantified using an OxyBlot Protein Oxidation Detection Kit (S7150, Merck, USA) according to the manufacturer’s instructions with minor modifications. Briefly, the ECM on plates were derivatized by incubating with 50 μL of 1 × DNPH for 15 min at 21 °C, then rinsed twice with PBS followed by blocking with 0.1% (w/v) casein in PBS. Plates were then incubated with rabbit anti-DNP antibody (1:500) at 4 °C overnight, rinsed twice with PBST (0.1% Tween-20 in PBS), incubated with horseradish peroxidase (HRP)-conjugated sheep anti-rabbit IgG secondary antibodies (1:1000) for 1 h at 21 °C, then washed twice with PBST. The plates were subsequently developed in 50 mM sodium citrate (pH 4.6) buffer containing 2 mM ABTS and 3 mM H_2_O_2_ for 30 min, with the resulting absorbance at 405 nm measured using a Spectra Max i3x microplate reader (Molecular Devices).

### Statistics

All the statistical analyses was performed by one-way ANOVA and post-hoc testing as described in the figure legends, using IBM SPSS 19.0. Results are representative of triplicate independent experiments (n = 3) with * and ** indicating statistical significance at the *p* < 0.05 and *p* < 0.01 levels respectively.

## Supplementary information

Supplementary information.

## References

[1] Wight, T. N. In *Extracellular Matrix* Vol. 1 (ed. Comper, W. D.) pp. 175–202 (Harwood Academic Publishers, 1996).

[2] Chuang CY, Degendorfer G, Davies MJ (2014). Oxidation and modification of extracellular matrix and its role in disease. Free. Radic. Res..

[3] Nissen R, Cardinale GJ, Udenfriend S (1978). Increased turnover of arterial collagen in hypertensive rats. Proc. Natl Acad. Sci. USA.

[4] Shapiro SD, Endicott SK, Province MA, Pierce JA, Campbell EJ (1991). Marked longevity of human lung parenchymal elastic fibers deduced from prevalence of D-aspartate and nuclear weapons-related radiocarbon. J. Clin. Invest..

[5] Halliwell B, Gutteridge JM (1990). The antioxidants of human extracellular fluids. Arch. Biochem. Biophys..

[6] Rees MD, Kennett EC, Whitelock JM, Davies MJ (2008). Oxidative damage to extracellular matrix and its role in human pathologies. Free. Radic. Biol. Med..

[7] Woods AA, Linton SM, Davies MJ (2003). Detection of HOCl-mediated protein oxidation products in the extracellular matrix of human atherosclerotic plaques. Biochem. J..

[8] Libby P, Ridker PM, Hansson GK (2011). Progress and challenges in translating the biology of atherosclerosis. Nature.

[9] Fischer GM, Llaurado JG (1966). Collagen and elastin content in canine arteries selected from functionally different vascular beds. Circ. Res..

[10] Fu S, Davies MJ, Stocker R, Dean RT (1998). Evidence for roles of radicals in protein oxidation in advanced human atherosclerotic plaque. Biochem. J..

[11] Stadler N, Lindner RA, Davies MJ (2004). Direct detection and quantification of transition metal ions in human atherosclerotic plaques: evidence for the presence of elevated levels of iron and copper. Arterioscler. Thromb. Vasc. Biol..

[12] Stanley N, Stadler N, Woods AA, Bannon PG, Davies MJ (2006). Concentrations of iron correlate with the extent of protein, but not lipid, oxidation in advanced human atherosclerotic lesions. Free. Radic. Biol. Med..

[13] Beckman JS (1994). Extensive nitration of protein tyrosines in human atherosclerosis detected by immunohistochemistry. Biol. Chem. Hoppe-Seyler.

[14] Leeuwenburgh C (1997). Reactive nitrogen intermediates promote low density lipoprotein oxidation in human atherosclerotic intima. J. Biol. Chem..

[15] Degendorfer G, Chuang CY, Hammer A, Malle E, Davies MJ (2015). Peroxynitrous acid induces structural and functional modifications to basement membranes and its key component, laminin. Free. Radic. Biol. Med..

[16] Degendorfer G (2016). Peroxynitrite-mediated oxidation of plasma fibronectin. Free. Radic. Biol. Med..

[17] Hazen SL, Heinecke JW (1997). 3-Chlorotyrosine, a specific marker of myeloperoxidase-catalyzed oxidation, is markedly elevated in low density lipoprotein isolated from human atherosclerotic intima. J. Clin. Invest..

[18] Hazell LJ (1996). Presence of hypochlorite-modified proteins in human atherosclerotic lesions. J. Clin. Invest..

[19] Hazell LJ, Baernthaler G, Stocker R (2001). Correlation between intima-to-media ratio, apolipoprotein B-100, myeloperoxidase, and hypochlorite-oxidized proteins in human atherosclerosis. Free. Radic. Biol. Med..

[20] Vanichkitrungruang S, Chuang CY, Davies MJ (2018). Oxidation of human plasma fibronectin by hypochlorous (HOCl) and hypothiocyanous (HOSCN) acids perturbs endothelial cell function. Free. Radic. Biol. Med..

[21] Takeshita J (2005). Myeloperoxidase generates 5-chlorouracil in human atherosclerotics tissue: A potential pathway for somatic mutagenesis by macrophages. J. Biol. Chem..

[22] Shao B, Pennathur S, Heinecke JW (2012). Myeloperoxidase targets apolipoprotein A-I, the major high density lipoprotein protein, for site-specific oxidation in human atherosclerotic lesions. J. Biol. Chem..

[23] Davies MJ, Hawkins CL, Pattison DI, Rees MD (2008). Mammalian heme peroxidases: from molecular mechanisms to health implications. Antioxid. Redox Signal..

[24] Daugherty A, Dunn JL, Rateri DL, Heinecke JW (1994). Myeloperoxidase, a catalyst for lipoprotein oxidation, is expressed in human atherosclerotic lesions. J. Clin. Invest..

[25] Malle E (2000). Immunohistochemical evidence for the myeloperoxidase/H_2_O_2_/halide system in human atherosclerotic lesions: colocalization of myeloperoxidase and hypochlorite-modified proteins. Eur. J. Biochem/FEBS.

[26] Baldus Stephan, Heeschen Christopher, Meinertz Thomas, Zeiher Andreas M., Eiserich Jason P., Münzel Thomas, Simoons Maarten L., Hamm Christian W. (2003). Myeloperoxidase Serum Levels Predict Risk in Patients With Acute Coronary Syndromes. Circulation.

[27] Nussbaum C, Klinke A, Adam M, Baldus S, Sperandio M (2013). Myeloperoxidase: a leukocyte-derived protagonist of inflammation and cardiovascular disease. Antioxid. Redox Signal..

[28] Tang WH (2009). Usefulness of myeloperoxidase levels in healthy elderly subjects to predict risk of developing heart failure. Am. J. Cardiol..

[29] Baldus S (2002). Spatial mapping of pulmonary and vascular nitrotyrosine reveals the pivotal role of myeloperoxidase as a catalyst for tyrosine nitration in inflammatory diseases. Free. Radic. Biol. Med..

[30] Kubala L (2013). The potentiation of myeloperoxidase activity by the glycosaminoglycan-dependent binding of myeloperoxidase to proteins of the extracellular matrix. Biochim. Biophys. Acta.

[31] Rees MD (2010). Myeloperoxidase-derived oxidants selectively disrupt the protein core of the heparan sulfate proteoglycan perlecan. Matrix Biol..

[32] Baldus S (2006). Heparins increase endothelial nitric oxide bioavailability by liberating vessel-immobilized myeloperoxidase. Circulation.

[33] Manchanda K (2018). MPO (Myeloperoxidase) Reduces endothelial glycocalyx thickness dependent on its cationic charge. Arterioscler. Thromb. Vasc. Biol..

[34] Zouaoui Boudjeltia K (2004). Oxidation of low density lipoproteins by myeloperoxidase at the surface of endothelial cells: an additional mechanism to subendothelium oxidation. Biochem. Biophys. Res. Commun..

[35] Stefanadis, C., Antoniou, C. K., Tsiachris, D. & Pietri, P. Coronary atherosclerotic vulnerable plaque: current perspectives. *J Am Heart Assoc* **6**, 10.1161/JAHA.117.005543 (2017). 10.1161/JAHA.117.005543 PMC5524044 28314799

[36] Teng N (2017). The roles of myeloperoxidase in coronary artery disease and its potential implication in plaque rupture. Redox Rep..

[37] Goldmann BU (2009). Neutrophil activation precedes myocardial injury in patients with acute myocardial infarction. Free. Radic. Biol. Med..

[38] Sugiyama S (2001). Macrophage myeloperoxidase regulation by granulocyte macrophage colony-stimulating factor in human atherosclerosis and implications in acute coronary syndromes. Am. J. Pathol..

[39] Döring Y, Drechsler M, Soehnlein O, Weber C (2015). Neutrophils in Atherosclerosis: From Mice to Man. Arterioscler. Thromb. Vasc. Biol..

[40] Nybo T (2018). Chlorination and oxidation of human plasma fibronectin by myeloperoxidase-derived oxidants, and its consequences for smooth muscle cell function. Redox Biol..

[41] Lu H, Hoshiba T, Kawazoe N, Chen G (2012). Comparison of decellularization techniques for preparation of extracellular matrix scaffolds derived from three-dimensional cell culture. J. Biomed. Mater. Res. A.

[42] Mayorca-Guiliani AE (2017). ISDoT: *in situ* decellularization of tissues for high-resolution imaging and proteomic analysis of native extracellular matrix. Nat. Med..

[43] Cai H, Chuang CY, Vanichkitrungruang S, Hawkins CL, Davies MJ (2019). Hypochlorous acid-modified extracellular matrix contributes to the behavioral switching of human coronary artery smooth muscle cells. Free. Radic. Biol. Med..

[44] Dypbukt JM (2005). A sensitive and selective assay for chloramine production by myeloperoxidase. Free. Radic. Biol. Med..

[45] Hawkins CL, Davies MJ (1998). Hypochlorite-induced damage to proteins: formation of nitrogen-centred radicals from lysine residues and their role in protein fragmentation. Biochem. J..

[46] Tiwari MK (2018). Early events in copper-ion catalyzed oxidation of α-synuclein. Free. Radic. Biol. Med..

[47] Forman HJ, Bernardo A, Davies KJA (2016). What is the concentration of hydrogen peroxide in blood and plasma? (vol 603, pg 48, 2016). Arch. Biochem. Biophys..

[48] Stocker R, Keaney JF (2004). Role of oxidative modifications in atherosclerosis. Physiol. Rev..

[49] Klebanoff SJ, Kettle AJ, Rosen H, Winterbourn CC, Nauseef WM (2013). Myeloperoxidase: a front-line defender against phagocytosed microorganisms. J. Leukoc. Biol..

[50] Vartio T (1989). Regular fragmentation of hydrogen peroxide-treated fibronectin. J. Biol. Chem..

[51] Pattison DI, Davies MJ (2001). Absolute rate constants for the reaction of hypochlorous acid with protein side chains and peptide bonds. Chem. Res. Toxicol..

[52] Pattison DI, Davies MJ (2006). Reactions of myeloperoxidase-derived oxidants with biological substrates: gaining insight into human inflammatory diseases. Curr. Med. Chem..

[53] Hawkins CL, Pattison DI, Davies MJ (2003). Hypochlorite-induced oxidation of amino acids, peptides and proteins. Amino Acids.

[54] Fuentes-Lemus E (2018). Aggregation of alpha- and beta- caseins induced by peroxyl radicals involves secondary reactions of carbonyl compounds as well as di-tyrosine and di-tryptophan formation. Free. Radic. Biol. Med..

[55] Nybo T (2019). Chlorination and oxidation of the extracellular matrix protein laminin and basement membrane extracts by hypochlorous acid and myeloperoxidase. Redox Biol..

[56] Noble RW, Gibson QH (1970). The reaction of ferrous horseradish peroxidase with hydrogen peroxide. J. Biol. Chem..

[57] Morris JC (1966). The acid ionization constant of HOCl from 5 °C to 35 °C. J. Phys. Chem..

