# Supplementary material for: Binding of myeloperoxidase to the extracellular matrix of smooth muscle cells and subsequent matrix modification
Source: Sci Rep. 2020 Jan 20;10:666. doi: 10.1038/s41598-019-57299-6 (PMC6971288; doi:10.1038/s41598-019-57299-6)
Supplement: Supplementary file 1 — Supplementary information. [file 41598_2019_57299_MOESM1_ESM.pdf]

Binding of myeloperoxidase to the extracellular matrix of smooth muscle cells and subsequent matrix modification

Huan Cai, Christine Y. Chuang, Clare L. Hawkins, Michael J. Davies *

Department of Biomedical Sciences, Panum Institute, University of Copenhagen, Copenhagen, Denmark

Supplementary Data

Abbreviations

ABTS, 2,2'-azino-bis (3-ethylbenzothiazoline-6-sulphonic acid; DOC, sodium deoxycholate; ECM, extracellular matrix; ELISA, enzyme-linked immunosorbent assay; GAG, glycosaminoglycan; HCASMC, human coronary artery smooth muscle cells; HCASMC-ECM, extracellular matrix derived from HCASMCs; mAb, monoclonal antibody; HOCl, the physiological mixture of hypochlorous acid and its anion; MPO, myeloperoxidase; pAb, polyclonal antibody; PBST, phosphate-buffered saline with Tween 20; TMB, 3,3',5,5'-tetramethylbenzidine.

* Corresponding author at: Department of Biomedical Sciences, Panum Institute, University of Copenhagen, Blegdamsvej 3, Copenhagen 2200, Denmark

E-mail address: davies@sund.ku.dk (M.J. Davies)

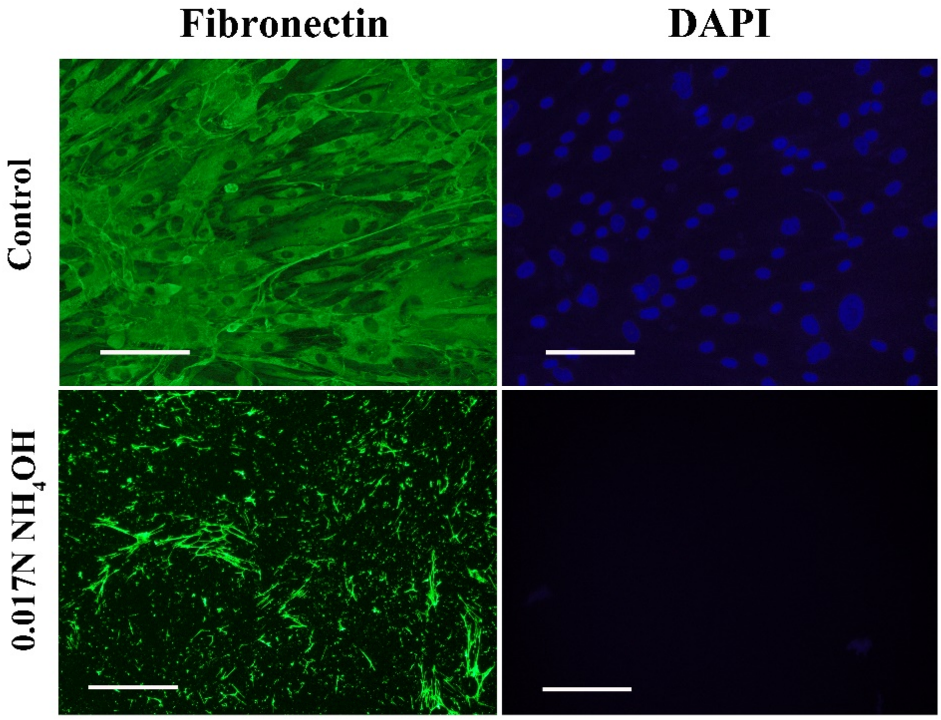

Supplementary Figure 1. Immunolocalization of components of HCASMC ECM prepared using ammonium hydroxide. HCASMCs were cultured for 1 week to allow synthesis of native ECM, followed by treatment without (top row) or with (bottom row) treatment with 0.017N ammonium hydroxide. Fibronectin was detected via immunofluorescence staining using anti-fibronectin pAb (**left panels**). An Alexa Fluor 488-conjugated anti-rabbit secondary antibody (green) was used to visualize fibronectin-primary antibody complexes, with nuclei counterstained using DAPI (blue, **right panels)**. Representative images from n= 3 independent experiments are shown. Scale bars: 50 µm.

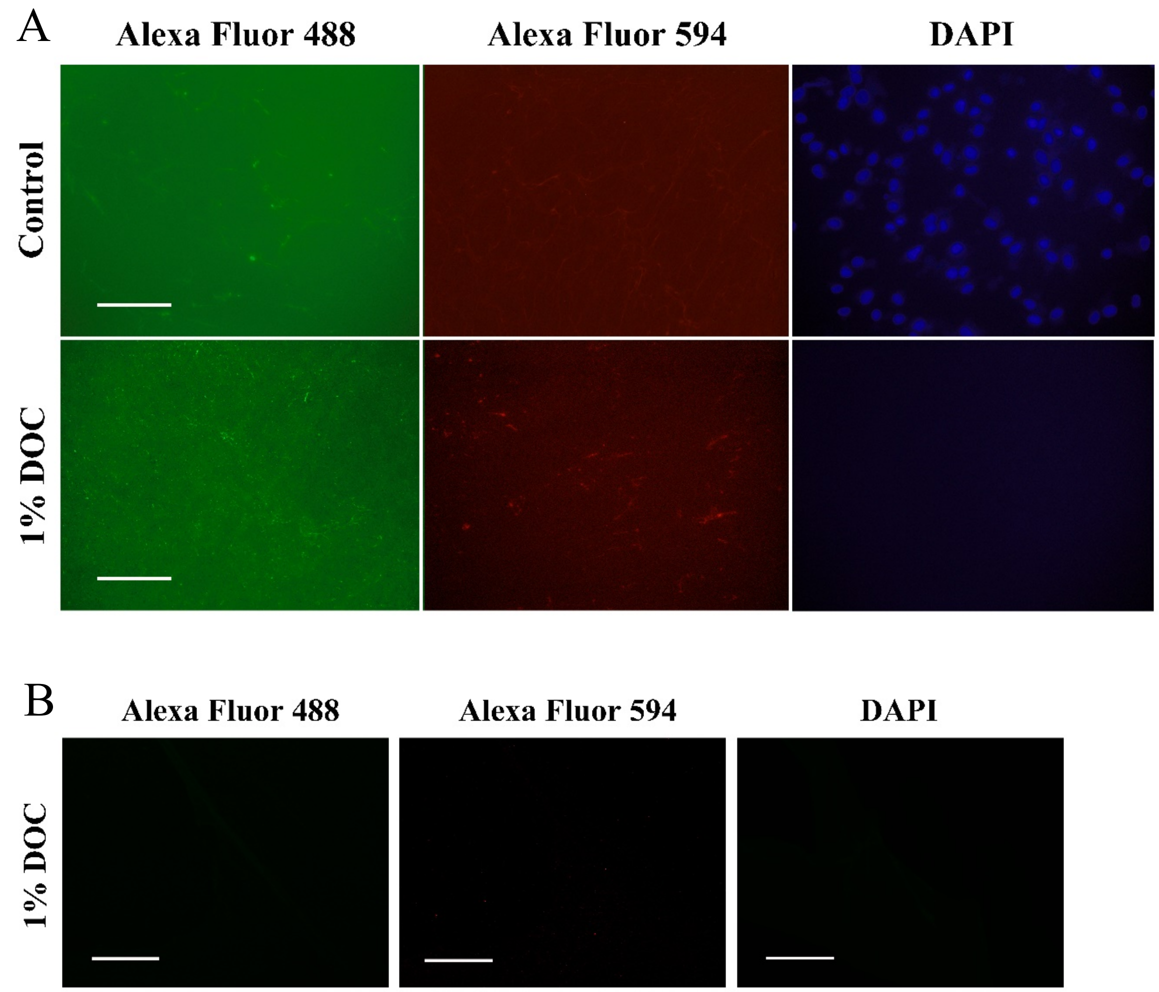

Supplementary Figure 2. Negative controls for immunofluorescence images. HCASMCs were cultured for 1 week to allow matrix synthesis. **(A)** HCASMC-ECM were either left untreated (control) or decellularized using 1% DOC to remove HCASMC, prior to incubation with fluorescently-tagged secondary antibodies to detect the presence of background fluorescence. **(B)** Decellularized HCASMC-ECM was exposed to 20 nM of MPO for 30 min at 37 ^{o}C prior to incubation with fluorescently-tagged secondary antibodies to detect the presence of background fluorescence for confocal microscopy. The secondary antibodies used were Alexa Fluor 488-conjugated anti-rabbit antibody (1:500 dilution; green) or Alexa Fluor 594-conjugated anti-mouse antibody (1:500; red). Nuclei were counterstained with DAPI

(1:1000; blue). Representative images from three independent experiments are shown (n=3).

Scale bars: 50 μm.

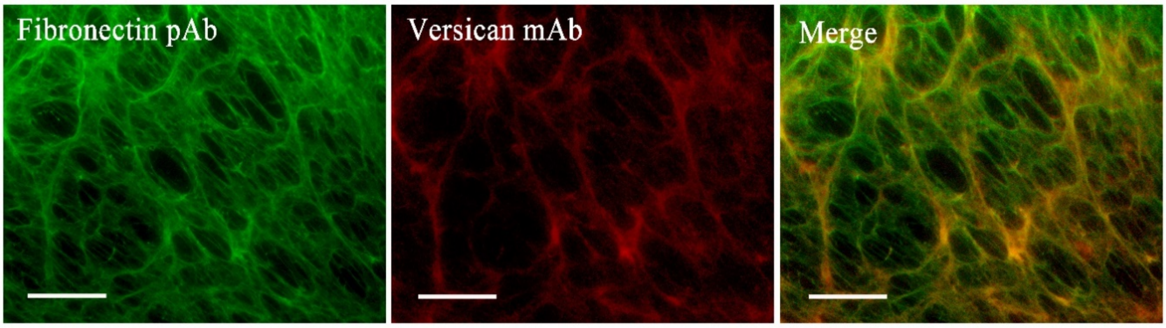

Supplementary Figure 3. Immunolocalization of decellularized ECM components synthesized by primary HCASMCs. HCASMCs were cultured for 1 week to allow matrices synthesis, followed by treatment with or without 1% sodium deoxycholate. Matrix proteins were detected via double immunofluorescence staining using anti-fibronectin pAb (**left panel**) and anti-versican G1 domain mAb (12C5) (**middle panel**). The merged image is shown in the **right panel**. An Alexa Fluor 488-conjugated anti-rabbit secondary antibody (green) and an Alexa Fluor 594-conjugated anti-mouse secondary antibody (red) were used to visualize matrix protein-primary antibody complexes. Representative images from n=3 independent experiments are shown. Scale bars: 50 µm.
